# Supplementary material for: Understanding healthcare providers’ perspectives on barriers to accessing stroke care at a resource-limited hospital in East Africa: A qualitative study from Mnazi Mmoja Referral Hospital in Zanzibar
Source: PLOS Glob Public Health. 2025 Feb 24;5(2):e0004278. doi: 10.1371/journal.pgph.0004278 (PMC11849830; doi:10.1371/journal.pgph.0004278)
Supplement: S1 Table — (DOCX) [file pgph.0004278.s001.docx]

| **Meaning unit** | **Code** | **Category** | **Theme** |
| --- | --- | --- | --- |
| The last three days I was on call I received the patient with massive hemorrhagic stroke, he died on arrival. He was convulsing since morning at 5 pm. He was…they keep him at home. They say it is mdudu. | Stroke caused by spiritual posession | Stroke as a supernatural condition | **Social representations and conceptualization of stroke** |
| In Swahili, if a person gets a stroke, his caretakers will think it is caused by spirits and give him herbal treatments.. |  |  |  |
| To some it is kazi ya mtu [witchcraft], to some it is not; everybody has their beliefs and understanding…some stick to what they believe. Even if you try to educate them they still believe what they believe. | Stroke caused by jelousy using witchcraft |  |  |
| Some, some will know they are hypertensive. If you ask the relatives ‘is your patient hyptertensive’ they will say yes. So this is the complication of it. But for those who dont know they are hypertensive and they get that suddently they directly belive its is ubaya [witchcraft]. |  |  |  |
| They think it is a spirit (...) In order for them to believe someone is getting sick maybe today [he is] not feeling well, tomorrow maybe having a headache, it will take 1-2-3 days for the symptoms to progress to that condition. That is why if it happens suddenly they dont believe that this patient is sick | Unaware of biomedical cause, progress and symptoms of stroke |  |  |
| The society has not gotten health education about stroke, they say here at ours in Zanzibar it is a disease caused by a spirit, you see. |  |  |  |
| Sometimes they bring a traditional healer to the ward, maybe at nightshift or evening shift when they know we are not there .. | Co-existence of beliefs |  |  |
| Yes because they dont use medication, they dont really believe that stroke has been caused by hypertension so they use herbal medication | Low treatment compliance |  |  |
| But when you pass to give him other medication he is not swallowing it, he can even hide it in the mouth and spit it out .. |  |  |  |
| Some doctors or nurses or orderlies tell the patients ‘According to what I see this patient should not be in hospital. This is [a problem that is concerned with] our spiritual world’. Something like that. Yes, but they are only few | HCPs also sharing supernatural beliefs |  |  |
| He might think about his family ‘I cannot walk any longer, who will take care of me, people will distance themselves from me (...) | Social isolation | Stroke as a devestating condition |  |
| It affects psychologically | Anxiety |  |  |
| He felt ashamed to have listened to his friends and used herbal medicine. It made him feel guilty | Guilt, shame |  |  |
| In general the challenge is that the stroke patient perhaps thinks his life has ended. That can affect psychologically. | Hopelessnesss |  |  |
| They say they get tired from taking the medication each and every day (...) but even psychologically I think they are affected, they loose hope. |  |  |  |
| They [HCPs] have already given up on him... | Poorer services |  |  |
| Because they don't believe it's a hospital-related matter. They say it is a spirit causing it. Therefore they are not taking good care of the patient. Because of this belief that it is not a hospital matter. | Stigma, discrimination |  |  |

**Table S1**: Example of thematic coding framework
