# Supplementary material for: Understanding healthcare providers’ perspectives on barriers to accessing stroke care at a resource-limited hospital in East Africa: A qualitative study from Mnazi Mmoja Referral Hospital in Zanzibar
Source: PLOS Glob Public Health. 2025 Feb 24;5(2):e0004278. doi: 10.1371/journal.pgph.0004278 (PMC11849830; doi:10.1371/journal.pgph.0004278)
Supplement: S2 Text — (DOCX) [file pgph.0004278.s002.docx]

**S2**: COREQ (Consolidated criteria for Reporting Qualitative research) Checklist

| **No** | **Item** | **Guide questions/description** | **Reported on page** |
| --- | --- | --- | --- |
|  | **Domain 1: Research team and reflexivity** |  |  |
|  | Personal characteristics |  |  |
| 1 | Interviewer/facilitator | Which author/s conducted the interview or focus group? | 6 |
| 2 | Credentials | What were the researcher’s credentials? E.g. PhD, MD | 6 |
| 3 | Occupation | What was their occupation at the time of the study? | 6 |
| 4 | Gender | Was the researcher male or female? | 6 |
| 5 | Experience and training | What experience or training did the researcher have? | 6 |
|  | Relationship with participants |  |  |
| 6 | Relationship established | Was a relationship established prior to study commencement? | 6 |
| 7 | Participant knowledge of the interviewer | What did the participants know about the researcher? e.g. personal goals, reasons for doing the research | 6 |
| 8 | Interviewer characteristics | What characteristics were reported about the inter viewer/facilitator? e.g. Bias, assumptions, reasons and interests in the research topic | 4+39 |
|  | **Domain 2: Study design** |  |  |
|  | Theoretical framework |  |  |
| 9 | Methodological orientation and Theory | What methodological orientation was stated to underpin the study? e.g.  grounded theory, discourse analysis, ethnography, phenomenology, content analysis | 4 |
|  | Participant selection |  |  |
| 10 | Sampling | How were participants selected? e.g. purposive, convenience, consecutive, snowball | 5 |
| 11 | Method of approach | How were participants approached? e.g. face-to-face, telephone, mail, email | 5 |
| 12 | Sample size | How many participants were in the study? | 7 |
| 13 | Non-participation | How many people refused to participate or dropped out? Reasons? | 6 |
|  | Setting |  |  |
| 14 | Setting of data collection | Where was the data collected? e.g. home, clinic, workplace | 8 |
| 15 | Presence of nonparticipants | Was anyone else present besides the participants and researchers? | 8 |
| 16 | Description of sample | What are the important characteristics of the sample? e.g. demographic data, date | 7 |
|  | Data collection |  |  |
| 17 | Interview guide | Were questions, prompts, guides provided by the authors? Was it pilot tested? | 5+8 |
| 18 | Repeat interviews | Were repeat inter views carried out? If yes, how many? | 8 |
| 19 | Audio/visual recording | Did the research use audio or visual recording to collect the data? | 8 |
| 20 | Field notes | Were field notes made during and/or after the interview or focus group? | 8 |
| 21 | Duration | What was the duration of the inter views or focus group? | 8 |
| 22 | Data saturation | Was data saturation discussed? | 6 |
| 23 | Transcripts returned | Were transcripts returned to participants for comment and/orcorrection | NO |
|  | **Domain 3: analysis and findings** |  |  |
| 24 | Data analysis |  |  |
| 25 | Number of data coders | How many data coders coded the data? | 8 |
| 26 | Description of the coding tree | Did authors provide a description of the coding tree? | Supplementary material |
| 27 | Derivation of themes | Were themes identified in advance or derived from the data? | 9 |
| 28 | Software | What software, if applicable, was used to manage the data? | 9 |
|  | Participant checking | Did participants provide feedback on the findings? | NO |
|  | Reporting |  |  |
| 29 | Quotations presented | Were participant quotations presented to illustrate the themes/findings?  Was each quotation identified? e.g. participant number | Yes, p 10 fw |
| 30 | Data and findings consistent | Was there consistency between the data presented and the findings? | yes |
| 31 | Clarity of major themes | Were major themes clearly presented in the findings? | Yes, p 10 fw |
| 32 | Clarity of minor themes | Is there a description of diverse cases or discussion of minor themes? | Yes, p 10 fw |
