# Supplementary material for: Understanding healthcare providers’ perspectives on barriers to accessing stroke care at a resource-limited hospital in East Africa: A qualitative study from Mnazi Mmoja Referral Hospital in Zanzibar
Source: PLOS Glob Public Health. 2025 Feb 24;5(2):e0004278. doi: 10.1371/journal.pgph.0004278 (PMC11849830; doi:10.1371/journal.pgph.0004278)
Supplement: S1 Text — (PDF) [file pgph.0004278.s003.pdf]

Interview guide v.1 | Stroke care providers | ZanStroke Study - substudy B

Introduction:

My name is Jutta Adelin Jorgensen, and I am working as a health researcher at a project on stroke called "ZanStroke". I'm interested in your experiences with and perspectives on stroke care, in particular which barriers you think there are in accessing stroke care in your setting . Participation to this interview is voluntary, and at any time during the interview, you can stop the conversation or skip questions. With your permission, I would like to record this conversation so that I can transcribe the interview, and your name will not be mentioned. After the end of the study the recordings will be deleted. The interview will take about half an hour. Do you have any questions before we begin?

Participant information:

Sex: *Age:* *Education and occupation:*

Employed at MMH since: *Working on this ward since :*

Broad category of question	***Follow up question***	***Probing***
**Tell me a bit about yourself and your work**	What is your role on this hospital/ward?<br><br>What do you enjoy the most about your job?	
**Can you please tell me about your experiences caring for people with stroke?**	What is expected you do when a patient arrives at this hospital/ward?	Any other things?
	What are the different types of tasks you do? [domains of tasks/activities]	Are there other tasks you do? Are there other tasks your colleagues do?
**Is providing stroke care different from 'ordinary' nursing care?**<br><br>**In your view, what is good stroke care?**<br><br>**Can you tell me about a stroke care situation where you were pleased with the care provided?**	Why / why not do you think it is different?<br><br>Can you give an example?<br><br>Who provides this care (team members)?<br><br>Was there anything special about this situation?	How?<br><br>Which elements in particular is making this an example of good care?<br><br>Are there more people involved in this care (relatives?)?<br><br>Why do you think it went well?
**Now I'm going to ask some differnt types of questions, and I'm interested in hearing your opinions**		
1) on how stroke patients recover. Could you tell me about that?		
2) What do you think impacts their recovery?	Can you name some aspects you think impact recovery?	Could you mention some more?
3) What about traditional/herbal/spiritual treatments ?	Are some treatments harmful / useful?	Which ones?
	Why?	Can you give examples?
	Which traditional treatments would you recommend patients to use, or would you use yourself? Which ones would you not use?	Why?
Before I asked you about what you find is good stroke care, and you told me about a situation you were involved in and where you felt content with the care given. Now I'm curious to hear about what you think **patients and their relatives** might think is good care -		
**In your view, what do patients and relatives feel is good care?**	What do you think is important to them?	Can you think of anything more?
	Is it easy for them to access this?	Why / why not?

**Have you encountered any dilemmas caring for patients with stroke?**	Which of these challenges are the biggest? Second biggest?	(if relevant then probe each of O/S, guidelines, manpower, time, knowledge, patients health understanding, colleagues etc)
	What do you feel about that?<br>Can you describe a dilemma you have been in?<br>Can the perspective of the patient, their relatives and the provider differ?<br>Is this an obstacle for providing good care?	What happened? How did you handle it?<br>How? can you give an example?<br>How?
**What makes a provider feel confident about providing stroke care?**	Can you give examples of elements that helps the provider to feel confident that he/she can do his/her job?	Can you think of anything else?
**What could make a provider feel insecure in providing stroke care?**	How, can you give examples?	
	Do you sometimes feel insecure in how to manage the patients?	How do you handle that?
Before I asked you about a situation where you were content with the care delivered. Now I'd like you to think about a situation where you were **not so content** with the care provided - it can be a situation you experienced yourself, or a situation you have heard about		
**Can you please tell me about a stroke care situation where you were *not so* content with the care provided?**<br>**Do you have another example where a provider would not be content?**	What happened? Have you any thoughts about why this happened?<br>Do you think it is a common situation?<br>What do you feel in this situation?	What else happened?<br>What can this lead to?
**What else - in your view - may challenge decisions to seek and access care when in hospital?**	[follow up questions]	
**Could you for a start mention some (other) obstacles to good care ?**	What do you think are the largest obstacles to good care here where you work?<br>[different types of barriers]<br>Who creates these obstacles / who is responsible for resolving them?	Are there other obstacles?<br>Examples?
**Do you think other health care workers would mention other barriers?**	Which ones? Examples<br>Do different cadres have different obstacles they face?	Why do you think they mention this?<br>Examples?
**What about traditional treatments and ideas about stroke - are they helping the patients?**	How? How not?<br>Do they affect if the patient receives good care?	Could you give an example?<br>How / how not?<br>Can you give some examples?
**Now I'm going to shift focus a bit, because I wonder how it is living in this culture (of traditional treatments and ideas) and a health professional. We spoke a little bit about that before ________________.**		
**I wonder if you have every experienced a conflict between what your patients think, and what you feel is the right to do as a health professional when it comes to acute stroke care?**		Can you give some examples?

**Are there also areas where it is a benefit to have 'other' ideas about stroke than what you learned at the health college?**	Can you give an example?	I would like to learn more about this. Can you tell me more about that?
**I have not more questions today, and thank you so much for teaching me about these things. But before we end today, I would like to hear if there is something I haven't asked you about that you think I should know?**		
