# Supplementary material for: Understanding healthcare providers’ perspectives on barriers to accessing stroke care at a resource-limited hospital in East Africa: A qualitative study from Mnazi Mmoja Referral Hospital in Zanzibar
Source: PLOS Glob Public Health. 2025 Feb 24;5(2):e0004278. doi: 10.1371/journal.pgph.0004278 (PMC11849830; doi:10.1371/journal.pgph.0004278)
Supplement: Attachment — Submitted filename: ResponseToReviewer.docx [file pgph.0004278.s004.docx]

Reviewer #1: Overall, this is an interesting paper and commend the authors for conducting this significant research in East Africa.

*Authors’ response:*

Thank you for taking time to review our manuscript, and for your valid comments and valuable suggestions throughout. In the below we have addressed each of the issues raised by you. We have avoided copying the amended text into our responses as the manuscript has undergone a major revision and text pieces would take up disproportionally large space; instead we refer to the manuscript with tracked changes.

Comments and suggestions for Authors:

Abstract

The abstract should be concise and well-organized, with a background, goal or objective, methodology, results, and a conclusion. The manuscript should be formatted consistently throughout.

The method section of the abstract and the manuscript must include the time and duration of the study. Also, authors should describe the theoretical approach or framework being used in the study if any.

*Authors’ response:*

Thank you for these suggestions. We have now included the time and duration of the study as well as the theoretical approach used. We have ensured the abstract is concise and well-organized with the recommended structure while sub-headings are not inserted as per PLOS Global Public Health house rules. Information on theoretical approach and timeline remains in the main text on pages 5 and 10.

Introduction :

The background should address the details of the previous study that had been done in the same institution and briefly describe any gaps in that research. This will eventually lead to the justification for the current study in the following paragraph, which will then lead to the primary objective of the research.

On page#3 it is indicated that that "the burden of stroke is expected to increase" (how much?). It would be more precise for readers to see the statistical data for the rate of incidence/prevalence and the mortality rate.

Before the section on the methodology, the study's objectives and purpose should be stated explicitly.

*Authors’ response:*

We thank you for this recommendation for the introduction which has improved the manuscript. We have now included statistical data for the incidence and mortality rate derived from the previous study in Zanzibar, described the knowledge gap identified which leads to an explicit description of the aim and specific objective of the current study. We define the term ‘barriers to healthcare access’ which is used throughout the manuscript.

Study design:

According to the COREQ checklist, it is recommended practice to include information regarding the type of sampling in the study design.

The characteristics and details of the study participants together with the participation selection and non-participation criteria must be included, such as years of experience working with stroke patients, etc. Also, authors may explicitly include the inclusion and exclusion criteria.

For authors reference; Tong A, Sainsbury P, Craig J. Consolidated criteria for reporting qualitative research (COREQ): a 32-item checklist for interviews and focus groups. International journal for quality in health care. 2007 Dec 1;19(6):349-57.

It is unclear whether the purposive sampling method or the snowball sampling method was the one used, therefore the information about the type of sampling that was performed must be included. Please carefully go through the information pertaining to the “informants”.

*Authors’ response:*

Thank you for this comment. Following the recommendations, we have re-written the section “Informants” page 7 to explicitly state the selection criteria used and reasons for the wide inclusion criteria. We have also clarified the sampling plan and recruitment plan used, including that “snowball sampling” is referring to the recruitment strategy.

We are also drawing the reviewer’s attention to the COREQ form in supplementary materials.

The description of the building, the wards, and the visiting hours in the study setting section don't seem to have any relevance specific to the objective of the current study. Authors may eliminate that information and replace it with more pertinent information about the study, including the location of the data collection etc.

*Authors’ response*

We thank the reviewer for this comment which enables us to elaborate on the use of ‘thick descriptions’ in our study.

In contrast to quantitative research, there are no set criteria to evaluate whether an interpretation in qualitative research is credible and truthful, and if it is better than other interpretations (Schwandt 2007). Rather, there are several thoughts about this, one being the concept of *trustworthiness* of research findings (Lincoln & Guba, 1985). Trustworthiness consists of the four interrelated criteria credibility, dependability, confirmability, and *transferability*. Transferability refers to the potential for findings to have relevance beyond the study’s specific participants and context. There is no specific strategy to ensure transferability, but by ensuring data saturation and providing thick descriptions of the study participants and context, including the study site, the reader should be able to assess if the findings can be transferred to another context. Therefore, thick descriptions is an important part of what enables the reader to asses the trustworthiness of a study.

In our study, data collection took place at a location chosen by the informant, and all informants chose this location to be at the hospital. Therefore, we have included some lines to give a thick description of the hospital. The interview typically took place at the nurses’ or doctors’ office, but sometimes a quiet corner of the green spaces outside the hospital was used.

Yet, in the revised manuscript we have slightly shortened the description of the physical setting and focused on how this is related to the data collection so the purpose of these thick descriptions is clear to the readers.

Data collection section and interview guide:

Authors should provide a justification for considering conducting a semi-structured interview.

As indicated, pilot research served as the basis for the interview guide's design. It will be valuable to provide more information regarding the pilot testing for the interview guide and how the questions designed for stroke patients and their families are equally pertinent to healthcare professionals interview guides. Please refer to Page #7.

*Authors’ response:*

Thank you for this comment. We have in this section of the manuscript now provided a justification for the approach and method used and included details on the pilot testing for the interview guide used in this study.

Regarding the reviewer’s comment ‘(…) how the questions designed for stroke patients and their families are equally pertinent to healthcare professionals interview guides’ we wonder if there is a small misunderstanding as we were not using questions designed for stroke patients and their families. Rather page 7 reads that the interview guide was designed to cover ‘(…) several domains, including professionalism, organization and management, human resources, and individual patient/family/health provider factors’. Patient/family factors are related to the population’s ability to perceive, seek, reach, pay and engage with the health system (Levesque 2012). With the revision of the background section to include description of Levesque’s conceptual framework of healthcare access this part should be free from misunderstandings in the revised manuscript.

The authors should provide the interview guide as an appendix or supplementary materials.

*Authors’ response:*

Thank you for this advice, we have now submitted the interview guide as supplementary material.

Analysis:

The authors must clarify precisely how the data in language other than English was handled during the coding and analysis process.

*Authors’ response:*

Thank you for pointing this out. We have now clarified in the ‘Analysis’ section how data in language other than English was handled including if and when translation of text took place, and by whom.

Page#31: Authors should define "Biomedical ideas about stroke" and "tension between professionals"?

*Authors’ response:*

Thank you for pointing this out. We have now re-written the parts and defined meanings when used.

Discussion:

Themes and subthemes have been repeated, and authors must address new insights on the current research in more detail in the discussion section.

The arguments presented with the scientific literature are unclear and occasionally contradictory. For instance, on page#35, authors suggested task sharing as one of the potential strategies, but in the subsequent phrase, disregard the strategy by citing data from other Tanzanian settings which demonstrated the limitation of the task sharing approach in such contexts.

*Authors’ response:*

Thank you for these useful comments. As the manuscript is rather lengthy and to assist the reader not to ‘get lost’ we find it useful to have the summary of identified themes and subthemes remain as an introduction to the discussion section. We have rewritten significant parts of the discussion section to discuss selected findings more in detail, including how our findings add to the existing knowledge base around barriers to stroke care access. Also, in the revised manuscript we aim to highlight the ambiguities that exist, for instance regarding task sharing, and that it is in this complex situation that context specific solutions must be found.

Second paragraph on page#37 The first line is extremely lengthy, incoherent, and lacks a clear meaning. What precisely is biomedical treatment? Does it imply receiving medical treatment?

*Authors’ response:*

Noted, and we have rewritten the paragraph.

Strengths and limitations:

Page#39: Authors must mention the details of the colleagues with whom the analysis and essential findings to limit cross cultural misinterpretations was discussed along with why and how the authors did it?

*Authors’ response:*

Thank you for this suggestion. We have included the requested details in the revised manuscript in the section ‘Analysis’.

While the study appears to be sound. The language is occasionally unclear, and sentences are too lengthy, and need to be rephrased to make them more concise and simpler to comprehend. I advise the authors work with the writing coach or editor to improve the flow and readability of the text.

*Authors’ response:*

Thank you for this valuable advice. Substantial parts of the manuscript have now been re-written, and we trust that the reviewer will find that the revised version provides a clear, coherent text.
