# Supplementary material for: Understanding healthcare providers’ perspectives on barriers to accessing stroke care at a resource-limited hospital in East Africa: A qualitative study from Mnazi Mmoja Referral Hospital in Zanzibar
Source: PLOS Glob Public Health. 2025 Feb 24;5(2):e0004278. doi: 10.1371/journal.pgph.0004278 (PMC11849830; doi:10.1371/journal.pgph.0004278)
Supplement: Attachment — Submitted filename: 2nd Response to Reviewer.docx [file pgph.0004278.s005.docx]

Response to Reviewer #1

Thank you for your positive feedback after our first revision in which you state that authors have satisfactorily addressed all your concerns.

There were no second reviewer on this submission.

We have emailed the editorial office several times to enquire if there are any revisions for us to work on, as there are no comments, concerns, or guidance on revisions needed when the manuscript was sent back to us.

As we have not received a response on needed revision, we consider the manuscript complete and ready to proceed.

If you have any questions or concerns please do not hesitate to contact us.

Warm regards,

Jutta Jorgensen
