# Supplementary material for: Understanding healthcare providers’ perspectives on barriers to accessing stroke care at a resource-limited hospital in East Africa: A qualitative study from Mnazi Mmoja Referral Hospital in Zanzibar
Source: PLOS Glob Public Health. 2025 Feb 24;5(2):e0004278. doi: 10.1371/journal.pgph.0004278 (PMC11849830; doi:10.1371/journal.pgph.0004278)
Supplement: Attachment — Submitted filename: 3rd response to reviewer.docx [file pgph.0004278.s006.docx]

**Authors responses to reviewer’s comments**

Thank you for carefully reviewing our manuscript and for the helpful comments given. The detailed responses to the issues raised are as follows:

**Explain the choice of semi-structured interviews over focus groups**

The choice to use semi-structured interviews were based on the sensitive nature of the topic (involving potential critique of the health system performance including of health professionals), the desire to capture diverse professional perspectives (being sensitive of the hierarchy among professionals that would make it necessary to conduct many FDG to capture their diverse perspectives without dominance of certain voices); and the interest in in-depth exploration of individual perspectives (that might not be fully expressed in a group setting due to peer influence or group dynamics, or concerns about judgement or repercussions). Semi-structured interviewing ensures privacy and confidentiality of the interviewee, and there is a general consensus in the qualitative research field that while FGD are valuable for exploring shared experiences and understanding cultural norms, they are less suited for sensitive or deeply personal topics where SSI are better suited (see for example Rosaline Barbour ‘Introducing qualitative research - a student’s guide’ (2008) or ‘Conducting Focus Group Discussions’ (2007) by same author).

We have added this to the manuscript text to now read as follows:

‘ Semi-structured interviews were chosen due to the sensitive nature of the topic, potential critique of the health system, and the need to capture diverse professional perspectives without hierarchical dominance. They allowed in-depth exploration of individual views, free from peer influence or fear of judgment, ensuring privacy and confidentiality. ‘ (page 5 - study design)

**Ensure that Table 2 aligns with the thematic structure in the text for consistency.**

Thank you for paying attention to this. We have carefully assessed and revised Table 2 that it aligns 1:1 with the thematic structure in the text including exact wordings in subheadings under each theme.

**Include a visual representation of the patient care pathway, highlighting barriers at each stage**

Thank you; Table 2 is in fact a visual representation of the patient care pathway (right hand side, vertical) with barriers (horizontally) present at each stage highlighted with X. We have revised the layout including changed colours to ease the interpretation and prevent misunderstandings.

**Strengthen the justification for purposive sampling**

Purposive sampling was selected to align closely with our study’s objective of understanding healthcare providers' perspectives on barriers to stroke care at Mnazi Mmoja Referral Hospital. This method ensured that participants were directly involved in stroke care and had recent, relevant, first hand experience to share.

A key strength of purposive sampling was that it allowed to select those with relevant experience while including participants from diverse professional backgrounds, roles, levels of seniority, and genders. This diversity allowed the study to encompass a wide range of perspectives to analyse barriers across various stages of stroke care. Our study applied a phenomenological approach and focused on participants with lived experiences of the phenomenon under investigation, ensuring that the findings are deeply rooted in real-world insights. Compared to random sampling, purposive sampling provided a more structured and strategic approach, and, we argue, enhanced the depth, relevance, and methodological rigor of the study.

In practical terms, purposive sampling was also an efficient approach for the low-budget study we conducted. It avoided the risk of including participants with little or no exposure to stroke care, which might dilute the depth and relevance of the data, and avoiding collecting ‘too much’ data, meaning an unnecessarily large dataset to transcribe and analyse - without providing additional information.

We have added this to the manuscript text to now read as follows:

‘Purposive sampling was chosen to align with the study's objective of understanding healthcare providers' perspectives on barriers to stroke care at Mnazi Mmoja Referral Hospital. It ensured the inclusion of participants with direct, recent, and relevant experience, and guaranteeing maximum variation by capturing diverse perspectives across professional roles, seniority levels, and genders (26). This approach, suited for phenomenological studies, focused on participants with lived experiences, ensuring findings were deeply rooted in real-world insights. It offered a structured, strategic alternative to random sampling, enhancing the study's depth, relevance, and rigor. Practically, purposive sampling was efficient for a low-budget study, avoiding irrelevant participants and unnecessary data collection, reducing transcription and analysis workload without compromising data quality.‘ (page 7 - informants)
