# Supplementary material for: Understanding healthcare providers’ perspectives on barriers to accessing stroke care at a resource-limited hospital in East Africa: A qualitative study from Mnazi Mmoja Referral Hospital in Zanzibar
Source: PLOS Glob Public Health. 2025 Feb 24;5(2):e0004278. doi: 10.1371/journal.pgph.0004278 (PMC11849830; doi:10.1371/journal.pgph.0004278)
Supplement: Attachment — Submitted filename: 4th Response to Reviewers.docx [file pgph.0004278.s007.docx]

**4^th^ Response to Reviewers**

Journal Requirements:

1. Please include a complete copy of PLOS’ questionnaire on inclusivity in global research in your revised manuscript.

*Response:*

A complete copy of PLOS’ questionnaire on inclusivity in global research was submitted 24th January 2024 as a supplementary file and is resubmitted with updated page numbers today

2. Please review your reference list to ensure that it is complete and correct.

*Response:*

We have reviewed the reference list and not found any retracted references; and corrected the incomplete references to adhere to the Vancouver style of referencing.

Editor Comments:

Kindly review PLOS Authorship guidelines and include native resident researchers who could have contributed to data acquisition for the research.

Authors are requested to read https://www.nature.com/articles/d41586-021-01795-1 on meaningful collaborations and abide by Rule 9: Be ethical and fair about publications and authorship

*Response:*

Thank you for notifying us about the request to read the linked career column which is behind Nature’s paywall.

We would like to draw the editor’s attention to the information that the first author JAJ has been living and working uninterrupted for 15 years in Zanzibar where the research took place. Furthermore, the first author worked in close collaboration with co-author SSS who is a native resident researcher and clinician in Zanzibar. SSS was not directly involved in data acquisition (way too often native researchers are limited to roles as data collectors); instead, she was instrumental in data processing and analysis.

The research was not funded by any institution and took place unpaid in the spare time of the researchers. The research study came about as a response to a need identified by health planners and managers in Zanzibar on actionable data around stroke (see ‘Stroke in Zanzibar’ by JMA Jorgensen, Copenhagen University Campus Print, 2023), data was collected, analysed and written up on site, and disseminated among stakeholders locally.

A detailed reflexivity statement has been added to the methods section, under study design, which now includes to read as follows:

Through a partnership between a non-native researcher (JAJ) and a native researcher (SSS) we embodied a balanced approach to qualitative inquiry, drawing on the strengths of both insider and outsider perspectives. The non-native researcher’s prolonged immersion provided familiarity with local norms while maintaining an external lens that facilitated access to potentially sensitive data. As noted in discussions on power dynamics in research (26), this outsider role can foster participant openness due to perceived neutrality. Conversely, the native researcher contributed essential cultural expertise and linguistic fluency, ensuring the research captures nuanced local realities. While not claiming to be ideal, this collaboration strengthened cultural validity and enhanced ethical engagement, acknowledging the critical need for equitable input from those rooted in the community in which the research took place.

Reviewer comments

(none)

While revising your submission, please upload your figure files to the Preflight Analysis and Conversion Engine (PACE) digital diagnostic tool, https://pacev2.apexcovantage.com/. PACE helps ensure that figures meet PLOS requirements. To use PACE, you must first register as a user. Registration is free. Then, login and navigate to the UPLOAD tab, where you will find detailed instructions on how to use the tool. If you encounter any issues or have any questions when using PACE, please email PLOS at figures@plos.org. Please note that Supporting Information files do not need this step.

*Response:*

We uploaded figure files to PACE after the initial submission and have done it again today.
